# Eliciting Clavulanic Acid Biosynthesis: The Impact of *Bacillus velezensis* FZB42 on the Metabolism of *Streptoyces clavuligerus* ATCC 27064

**DOI:** 10.3390/metabo15050337

**Published:** 2025-05-19

**Authors:** Luisa F. Patiño, Carlos Caicedo-Montoya, Laura Pinilla-Mendoza, Jaison H. Cuartas, Rigoberto Ríos-Estepa

**Affiliations:** 1 Grupo de Bioprocesos, Departamento de Ingeniería Química, Universidad de Antioquia UdeA, Calle 70 No. 52–21, Medellín 050010, Colombia; luisa.patinoc@udea.edu.co (L.F.P.); candres.caicedo@udea.edu.co (C.C.-M.); laura.pinilla@udea.edu.co (L.P.-M.); 2 Independent Researcher, Medellín 050010, Colombia; jhcuarta@unal.edu.co; 3 Grupo de Investigación en Simulación, Diseño, Control y Optimización de Procesos (SIDCOP), Departamento de Ingeniería Química, Universidad de Antioquia, Medellín 050010, Colombia

**Keywords:** transcriptome, oxidative stress, RNA-Seq, microbial interaction, antibiotic regulation

## Abstract

**Background/Objectives:** Clavulanic acid (CA) is produced by cell suspension cultures of *Streptomyces clavuligerus* ATCC 27064, and is widely used as a beta-lactamase inhibitor to combat antibiotic resistance. CA titers are moderate due to bioprocess complexity, prompting ongoing efforts to overcome these limitations. **Methods:** In this study, we aimed to evaluate the effect of live and inactivated *Bacillus velezensis* FZB42 cells on CA production in *S. clavuligerus*, and to explore the transcriptional response underlying this interaction using RNA-seq technology. **Results:** The addition of dead and live cells of *B. velezensis* improved CA production by 1.4 and 2.0-fold, respectively. Furthermore, the transcriptome of *S. clavuligerus,* obtained with live cells of *B. velezensis* FZB42 at the peak of maximum CA production, revealed that 410 genes were up-regulated and 594 were down-regulated under these conditions, with a *padj* < 0.05. Most of the genes from the cephamycin C and CA clusters were up-regulated, which correlates well with the increase in CA production. Likewise, *S. clavuligerus* ATCC 27064 enhanced the expression of genes encoding enzymes that scavenge endogenous H_2_O_2_, as well as other genes related to oxidative stress defense. Regarding downregulated genes, we found that *S. clavuligerus* decreased the expression of genes involved in the biosynthesis of terpenoids, polyketides, and lantibiotics, as well as the expression of the operon involved in the synthesis of the pyrroloquinoline quinone (PQQ) cofactor. **Conclusions:** These findings contribute to the understanding of *S. clavuligerus* metabolism and pave the way for future metabolic engineering efforts aimed at obtaining CA-overproducing strains.

## 1. Introduction

Improving CA production in *S. clavuligerus* is crucial given its key role as a beta-lactamase inhibitor. Larger production of CA would significantly reduce manufacturing costs, thus enlarging availability for vulnerable communities who suffer from resistant bacterial infections. CA has a relatively limited market availability and a middle–to–high cost for the health system, especially in developing countries. The cost of CA is mainly related to the complexity of the production process and the current uncertainties about the regulatory elements controlling the CA biosynthetic gene cluster. Many efforts in bioprocess optimization [1] genetic and metabolic engineering [2,3], and separation and purification studies have been completed in an attempt to overcome low productivity. Nonetheless, low titers (~1–2 g/L) are still obtained when using a wild-type *S. clavuligerus* strain for CA production.

*S. clavuligerus* is well known for its ability to produce approximately 48 secondary metabolites, including a wide range of specialized compounds such as the beta-lactamase inhibitory protein (BLIP) [3], CA, and the β-lactam antibiotic cephamycin C, as well as holomycin, polyketides, terpenoids, and lantibiotics [4]. CA and cephamycin C are synthesized from the same supercluster and are positively co-regulated by the transcriptional activator CcaR, which binds to the promoter regions of both gene clusters [5]. In addition, *S. clavuligerus* harbors multiple genes encoding beta-lactamases, some of which are inducible in the presence of β-lactam antibiotics, suggesting a complex regulatory network involving both biosynthesis and resistance [6]. The production of CA and cephamycin C is strongly influenced by nutrient limitation, particularly low levels of phosphate and nitrogen, as well as by oxygen availability and redox balance [1]. Understanding how these environmental factors interact with regulatory pathways is essential for enhancing the biosynthetic potential of this organism.

The use of co-cultures as a strategy to induce or enhance the production of secondary metabolites has been successfully demonstrated in various *Streptomyces* species. Contact with cells or cell fragments from fungi, bacteria, or even pathogenic microorganisms can act as an elicitor, enhancing the synthesis of natural metabolites or inducing their production in *Streptomyces* [7]. For example, the co-culture of *S. coelicolor* with *Myxococcus xanthus* has been employed to elicit the production of actinorhodin. In this case, competition between the two species reduces iron availability in the culture medium, thereby activating the actinorhodin biosynthetic gene cluster [7]. These findings support the idea that microbial interactions, whether competitive or cooperative, can serve as powerful modulators of secondary metabolism in actinomycetes.

*B. velezensis* is an aerobic, soil-dwelling, Gram-positive, endospore-forming bacterium that promotes plant growth and exhibits strong antimicrobial activity. It contains a significant number of genes encoding secondary metabolites involved in both plant defense stimulation and microbial competition [8]. More specifically, *B. velezensis* displays a high genetic capacity for synthesizing cyclic lipopeptides (e.g., surfactin, bacillomycin D, fengycin, and bacillibactin) and polyketides (e.g., macrolactin, bacillaene, and difficidin) [9]. These compounds not only contribute to induced systemic resistance (ISR) in plants but also participate in microbial antagonism, redox modulation, iron competition, and signaling processes that can affect neighboring microorganisms [9]. Furthermore, *B. velezensis* harbors genes encoding beta-lactamases, which may allow it to counteract the effects of β-lactam-producing bacteria such as *Streptomyces* [10]. The biosynthesis of these metabolites is regulated by global transcriptional regulators, including Spo0A, ComA, and DegU, which respond to environmental cues such as nutrient availability, population density, and microbial contact [10]. In this context, *B. velezensis* represents a metabolically versatile soil bacterium capable of influencing the secondary metabolism of *S. clavuligerus*, potentially eliciting CA production through interspecies chemical communication.

In the present study, we evaluated the effect of live and inactivated cells of *B. velezensis* FZB42 on the production of CA in *S. clavuligerus* ATCC 27064. Given the broad metabolic repertoire of *B. velezensis* and its potential to engage in interspecies chemical signaling, we hypothesized that co-cultivation could modulate the metabolic activity of *S. clavuligerus*. To investigate this, we conducted co-culture experiments and analyzed the transcriptional response of *S. clavuligerus* under these conditions using RNA-seq. This study aims to provide insight into how microbial interactions can influence CA production and to identify candidate genes and regulatory pathways involved in the activation of CA biosynthesis.

## 2. Materials and Methods

### 2.1. Bacterial Strains and Media

*S. clavuligerus* ATCC 27064 was grown in GYM-Agar at 28 °C for 10 to 15 days, for spore production [11]. The spores were scraped off the agar surface using a sterile loop and resuspended in sterile deionized water. After filtering through cotton wool, the spore suspension was serially diluted to 10^9^ CFU mL^−1^ and stored in 20% glycerol at −80 °C. The *S. clavuligerus* spore’s suspension cultures were grown during 36 h (OD600 of 7) in trypticase soy broth (TSB) at 28 °C and 220 rpm in a rotary shaker. Mycelium cultures were stored in 20% glycerol at −80 °C.

A seed medium was used for *S. clavuligerus* culture at pH 6.8; its composition was as follows (g/L): glycerol, 15; peptone, 10; malt extract, 1.0; MgSO_4_, 0.75; MnCl_2_, 0.0001; FeSO_4_, 0.001; ZnSO_4_, 0.001; MOPS, 21; K_2_HPO_4_, 0.8. For CA production the soy protein isolate (ISP) medium was used with the following composition (g/L): glycerol, 30; soy protein isolate, 25; K_2_HPO_4_, 0.8; MgSO_4_·7H_2_O, 0.75; MnCl_2_·4H_2_O, 0.0001; FeSO_4_·7H_2_O, 0.001; ZnSO_4_·7H_2_O, 0.001; MOPS, 21; and pH fixed at 6.8 [12]. The pre-culture medium had the same composition as the culture medium, except for the glycerol concentration, which was adjusted to 15 g/L. All *S. clavuligerus* cultures were carried out in 250-baffled Erlenmeyer flasks containing 50 mL of medium. Pre-culture flasks were inoculated with the seed medium (10% *v*/*v*), and the culture medium was inoculated with 10% *v*/*v* of pre-culture medium. Cultures were incubated for 96 h, at 28 °C and 220 rpm.

*B. velezensis* FZB42 was cultured in Luria broth medium (LB) (Merck, Rahway, NJ, USA) at 37 °C and 150 rpm until reaching the exponential growth phase. Afterwards, it was stored in 20% glycerol at −80 °C.

### 2.2. Co-Culture Preparation

Co-cultures of *S. clavuligerus* with *B. velezensis* were carried out in ISP liquid media. The seed medium was inoculated with 1 mL of *S. clavuligerus* spores (for 24 h, at 28 °C and 220 rpm). Afterwards, the seed medium (5 mL) was transferred to 45 mL of pre-culture medium and incubated under the same operating conditions. For CA production, the ISP culture medium was inoculated with the pre-culture medium at 10% *v*/*v*.

For all experiments, *B. velezensis* was grown in LB for 20 h (OD_600nm_ between 2 and 3). The *B. velezensis* cells were centrifuged at 13,000 rpm for 10 min, and the cell pellet was washed and resuspended with sterile water in an equal volume. Following, the cell suspension was induced for CA production in the fermentation medium at 2% and 4% (*v*/*v*). Additionally, *B. velezensis* culture media filtrate and the dead cells were evaluated as plausible CA production inducers, as follows: the supernatant of the centrifuged *B. velezensis* vegetative cells was sterilized using a 0.22-μm pore size filter and added to the CA production medium. For dead cells, after centrifuging, the cultured cells were washed twice and resuspended in sterile water in an equal volume of boiling water for 30 min. Subsequently, the cellular viability of these cells was assessed by plating the previously boiled cells onto LB agar medium and incubating them for 7 days to ensure that this process was sufficient to kill them. For each potential inducer, either culture supernatant, living or dead cell suspensions, were added to the CA fermentation medium at 2% and 4% (*v*/*v*) along with *S. clavuligerus*.

Two control experimental designs were carried out to evaluate CA production. The first control experiment counted for inoculated *S. clavuligerus* without any elicitor; a second one consisted of pure LB medium in the same proportions of the elicitors (1%, 2%, and 4% (*v*/*v*)). All experiments were carried out in triplicate.

### 2.3. Analytical Techniques

Culture samples were centrifuged at 14,000× *g* for 10 min at 4 °C, and filtered through a 0.22 µm pore membrane. CA was determined by HPLC Agilent 1200 (Agilent Technologies, Waldbrom, Germany) equipped with a Diode Array Detector (Agilent Technologies, Palo Alto, CA, USA) at 312 nm, using a reverse phase ZORBAX Eclipse XDB-C_18_ (4.6 × 150 mm, 18 μm Agilent Technologies, Palo Alto, CA, USA) column; 96% *v*/*v* KH_2_PO_4_ (50 mM, pH 3.2); and a 6% *v*/*v* methanol solution was used as mobile phase at 1 mL/min. CA was imidazole-derivatized at a ratio 1:3; the reaction was kept at 28 °C for 15 min [13]. The glycerol concentration was determined by HPLC Agilent 1200 (Agilent Technologies, Waldbrom, Germany) equipped with a refractive Index Detectors (Agilent Technologies, Palo Alto, CA, USA), using a ICSep Coregel-87H3 Column (7.8 × 300 mm, Agilent Technologies, Palo Alto, CA, USA), and a 0.01N H_2_SO_4_ solution as mobile phase at 0.6 mL/min [13].

### 2.4. RNA Sequencing (RNA-Seq)

RNA extraction was performed only for the co-culture treatment that induced the highest CA production. Total RNA was extracted from cultures at 72 h of cultivation, when CA concentration was the highest out of the tested conditions. A 2 mL sample was centrifuged at 10,000× *g* for 15 min at 4 °C; the supernatant was stored for downstream analysis; cell pellets were resuspended in 800 μL of Monarch RNA protector 1× (Monarch^®^) and immediately stored at −80 °C for subsequent RNA extraction. Total RNA was extracted from fresh frozen cell pellets using QIAGEN RNeasy Plus Universal mini kit following manufacturer’s instructions (QIAGEN^®^, Germantown, MD, USA). The RNA samples were quantified using Qubit 2.0 Fluorometer (Thermo Fisher Scientific, Waltham, MA, USA), and RNA integrity was checked with 4200 TapeStation (Agilent Technologies, Palo Alto, CA, USA). Only samples with high-quality RNA (RNA integrity number ≥ 7.0) were used in the following mRNA library preparation. For library preparation and sequencing, samples were initially treated with TURBO DNase (Thermo Fisher Scientific, Waltham, MA, USA) to remove DNA contaminants. The next steps included performing rRNA depletion using QIAseq^®^ FastSelect™−rRNA Bacteria (QIAGEN^®^, Germantown, MD, USA), which was conducted following the manufacturer’s protocol. The strand-specific RNA sequencing library was prepared using the NEBNext Ultra II Directional RNA Library Prep Kit for Illumina, following the manufacturer’s instructions (NEB, Ipswich, MA, USA). The sequencing library was validated on the Agilent TapeStation (Agilent Technologies, Palo Alto, CA, USA) and quantified using Qubit 2.0 Fluorometer (Thermo Fisher Scientific, Waltham, MA, USA) as well as by quantitative PCR (KAPA Biosystems, Wilmington, MA, USA). The sequencing libraries were multiplexed and clustered on one lane of a flow cell. After clustering, the flow cell was loaded onto the Illumina Hiseq^TM^ 2000 (Illumina, San Diego, CA, USA) according to the manufacturer’s instructions. The samples were sequenced using a 2 × 150 bp paired-end (PE) configuration and ~350 M PE reads.

### 2.5. Data Processing

Quality control of the FASTQ format raw data was checked using FastQC then the raw data were trimmed by removing adapter sequences and reads with low quality, using the software Fastp (v0.23.4) [14]. The high-quality reads were used in subsequent alignments. The sequencing paired-end reads were mapped to the corresponding reference genome sequence (genome accession numbers for *Streptomyces clavuligerus* ATCC 27064, NZ_CP027858.1 and NZ_CP027859.1, chromosome and megaplasmid, respectively, and NC_009725.2 for *B. velezensis* FZB42) using Bowtie2 (Version 2.5.1) [15].

A mapped read was exported as a BAM file format using Samtools [16]. Aligned sequencing reads were counted using HTseq [17] based on all annotated genomic features of the *S. clavuligerus* reference genome (NCBI genome assembly GCF_005519465.1) and *B. velezensis* (NCBI genome assembly GCF_000015785.2). The DESeq2 package was used to normalize the data and identify differentially expressed genes; only genes with a *p* < 0.05 and adjusted *p*-value (*padj*) ≤ 0.05 were considered statistically significant and retained for further analysis [18]. The raw sequencing data were deposited in the SRA database of NCBI under the accession number PRJNA1125460.

### 2.6. Statistical Analysis

The experimental results were statistically analyzed using the R Statistical software (version 4.3.2) [19]. The data were subjected to analysis of variance (ANOVA) and Tukey’s HSD test. The significance threshold was set to *p* < 0.05.

## 3. Results

### 3.1. Effect of the Co-Culture on the Biosynthesis of Clavulanic Acid

**Figure 1:**
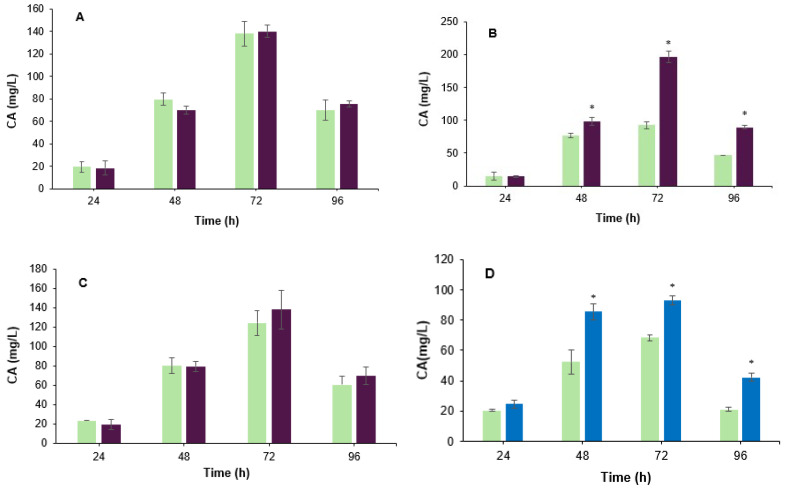
Production of CA by *S. clavuligerus* in a co-culture with live cells of *B. velezensis*. Color code: co-culture assay (purple); control assay (green). (**A**) 1% *v*/*v*. (**B**) 2% *v*/*v*. (**C**) 4% *v*/*v*. (**D**) CA production in the presence of 2% *v*/*v B. velezensis* dead cells. Color code: dead cells assay (blue); control (green). * Asterisks indicate that there is a significant difference.

Co-fermentation induces microbial competition for resources [20]. To evaluate the effect of *B. velezensis* biomass on CA biosynthesis, live cells (Figure 1A–C) and boiled cells (Figure 1D) were added separately to the fermentation broth along with *S. clavuligerus* ATCC 27064. As observed in Figure 1B, CA production was significantly (*p* < 0.05) larger when 2% *v*/*v* of *B. velezensis* live cells were added to the fermentation broth of *S. clavuligerus* ATCC 27064. Specifically, the CA yield increased 2.0-fold (197 mg/L) compared to the control assay (93 mg/L) after 72 h of cultivation. The addition of live cells at 4% caused a slight improvement of CA production (Figure 1C); this enhancement was not significant (*p* > 0.05). When live cells were added to the culture medium (1% *v*/*v*) (Figure 1A), no significant CA production changes (*p* > 0.05), compared to the control, were observed. In contrast, the addition of boiled cells to the culture medium (2% *v*/*v*) revealed a positive effect on CA production; compared to the control assay, the increment in CA yield was 1.6-fold and 1.4-fold at 48 h and 72 h of cultivation, respectively (Figure 1D). The ability of the boiled vegetative cells to grow was assessed on LB agar, and no growth was observed after 7 days of incubation, confirming that the treatment was effective. Although the contribution of released nutrients from dead *B. velezensis* cells cannot be excluded, subsequent transcriptomic and mechanistic analyses focused exclusively on the co-culture with live cells. The addition of live cells at 2% (*v*/*v*) was used as an elicitor for further analysis due to their higher enhancement effect.

### 3.2. Effect of B. velezensis Cell Filtrate on Clavulanic Acid Biosynthesis in S. clavuligerus

**Figure 2:**
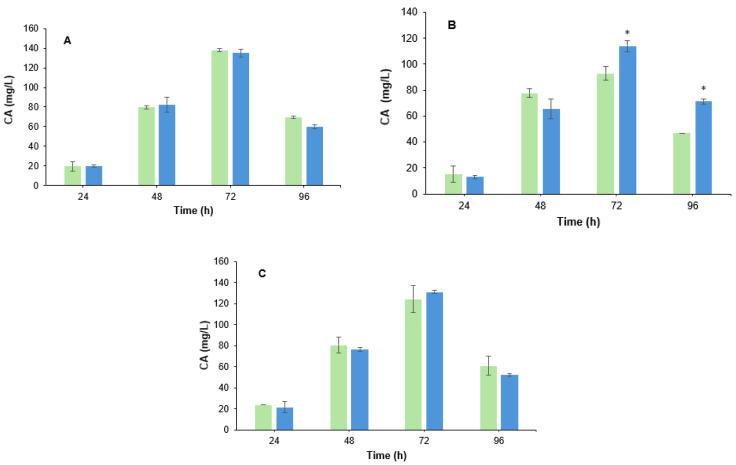
Production of CA by *S. clavuligerus* in the presence of filtrate from live cells. Color code: co-culture with filtrate (blue); control assay (green). (**A**) 1% *v*/*v*. (**B**) 2% *v*/*v*. (**C**) 4% *v*/*v*. * Asterisks indicate that there is a significant difference.

To determine the effect of the *B. velezensis* cells culture medium filtrate on CA biosynthesis, the live cells filtrate at 4%, 2%, and 1% (*v*/*v*) was added separately to the *S. clavuligerus* fermentation broth at 0 h, and the production of CA was measured over time. As revealed in Figure 2, a slight increase (1.2-fold, *p* < 0.05) in CA yield was observed at 72 h and 96 h (Figure 2B). However, when the filtrate was added at 4% *v*/*v* and 1% *v*/*v*, there were no significant differences in CA production between the co-cultured with filtrate and the control assay (Figure 2A,C).

### 3.3. Differential Expression Analysis

To further illustrate the transcriptional behavior of *B. velezensis* during co-culture with *S. clavuligerus* at 72 h, we provide a supplementary table (Supplementary Table S1) listing the Top 20 most differentially expressed genes based on their log_2_ fold change relative to control samples. This analysis is exploratory in nature, as it was derived from a single transcriptome without biological replicates, and therefore lacks formal statistical support. Nevertheless, it highlights key biological processes potentially affected in *B. velezensis* under the tested conditions, including motility repression, stress response activation, and iron homeostasis regulation (Supplementary Table S1).

**Figure 3:**
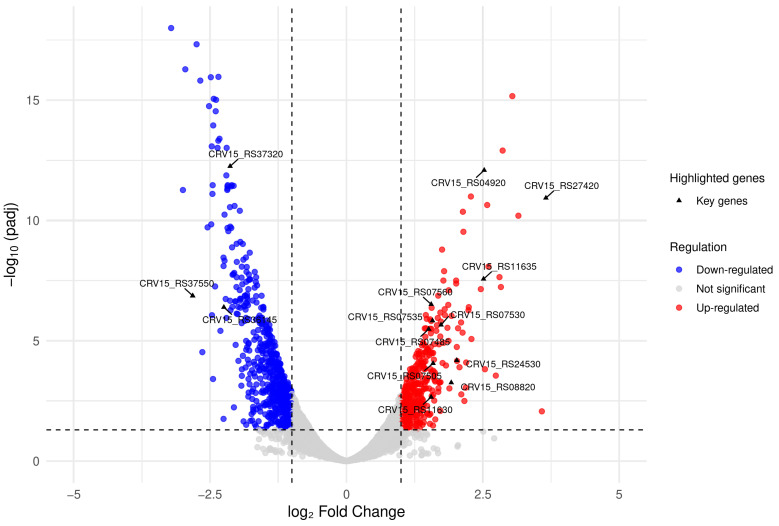
Volcano plot showing the differential gene expression in *S. clavuligerus* co-cultured with *B. velezensis*. Genes with log_2_ fold change > 1 or < −1 and adjusted *p*-value < 0.05 were considered significantly up-regulated (red) or down-regulated (blue), respectively. Genes not meeting these thresholds appear in light gray. The arrow indicates key genes discussed in the manuscript, such as *CRV15_RS07560 (claR)*, *CRV15_RS07530 (ceaS2)*, and others involved in CA biosynthesis or oxidative stress response, are highlighted for reference.

In parallel, to investigate how the *S. clavuligerus* cellular processes were affected during co-culture using 2% *v*/*v* of *B. velezensis* live cells, we performed an RNA-Seq analysis with the total RNA isolated from cells harvested at the highest CA production peak (72 h). Among the 6997 annotated genes of *S. clavuligerus* ATCC 27064, we observed 410 up-regulated genes (~6%) and 594 down-regulated genes (~9%), when comparing the pure-culture and co-culture conditions of *S. clavuligerus*, with a *padj* < 0.05 (Figure 3). Representation of the KEGG annotations for the differentially expressed genes is shown in Figure S1. Supplementary Material S1 and S2 show the DEGs with their corresponding fold change.

### 3.4. Gene Set Enrichment Analysis During Co-Culture

All protein-coding genes were processed using the BlastKOALA tool from the KEGG database (Kyoto Encyclopedia of Genes and Genomes) [21], for the purpose of gaining insights about how the up-regulated and down-regulated genes, or their gene products, contribute to different pathways or metabolic reactions. The analysis showed that, out of the 410 up-regulated genes, 226 were annotated; the majority of them were related to the production of proteins, DNA replication and repair, carbohydrate metabolism, amino acids and cofactors, as well as in the biosynthesis of secondary metabolites (Supplementary Figure S1). Regarding the down-regulated genes, only 292 out of 594 genes were annotated. Unlike the up-regulated genes, it can be seen that the down-regulated genes are related to the metabolism of lipids, terpenoids, polyketides, and ABC transporters (Supplementary Figure S1).

**Figure 4:**
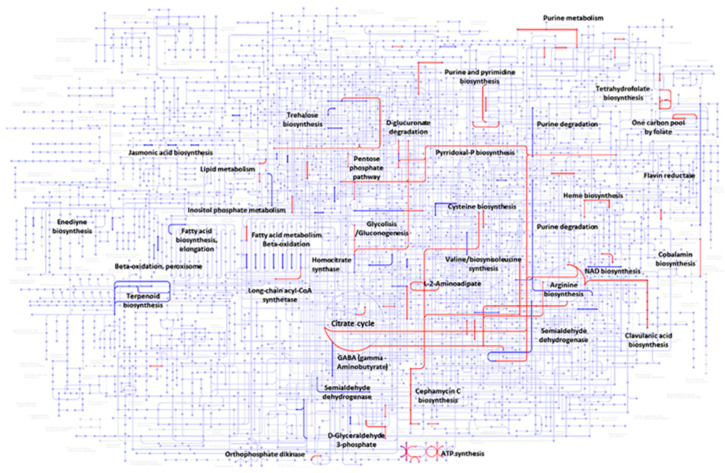
Metabolic pathways of S. *clavuligerus* (KEGG database). Color code: enzymes encoded by the up-regulated genes (red); enzymes encoded by the down-regulated genes (blue).

**Figure 5:**
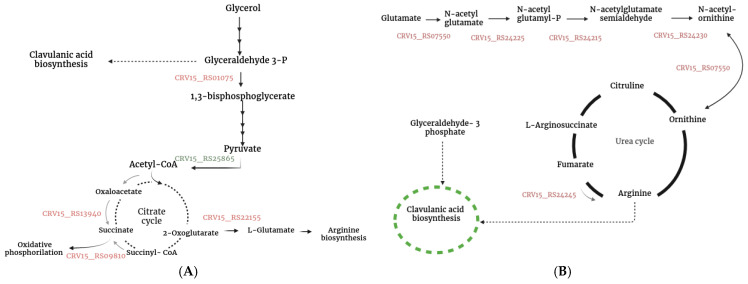
Up-regulated genes (in red) that encode enzymes related to the biosynthesis of CA precursors (glyceraldehyde 3-P and arginine). (**A**) Glycerol uptake; (**B**) L-arginine biosynthesis.

Figure 4 illustrates the metabolic pathways involving some of the up- and down-regulated genes annotated in KEGG. As observed, proteins encoded by up-regulated genes (highlighted in red) participate in the citric acid pathway, purine and pyrimidine biosynthesis, as well as in CA and cephamycin C biosynthesis. Explicitly, for CA biosynthesis, some proteins involved in the biosynthesis of L-arginine and D-glyceraldehyde 3-phosphate (G3P) (C-5 and C-3 CA precursors, respectively) were identified. For *S. clavuligerus*, glycerol is the main substrate that is incorporated into the glycolytic pathway, thus forming G3P [22]. Part of the G3P is directed to the CA pathway, while the rest continues through the glycolytic pathway until reaching the tricarboxylic acid cycle (TCA). Subsequently, L-glutamate is synthesized by transamination of 2-oxoglutarate (an intermediate of the TCA cycle), by glutamate synthase encoded by the gene CRV15_RS22155 (Figure 5A). Then, the C-5 CA precursor, L-arginine, is synthesized from L-glutamate through the urea cycle (Figure 5B); a fraction of it is used as the C-5 precursor of CA biosynthesis [23].

**Figure 6:**
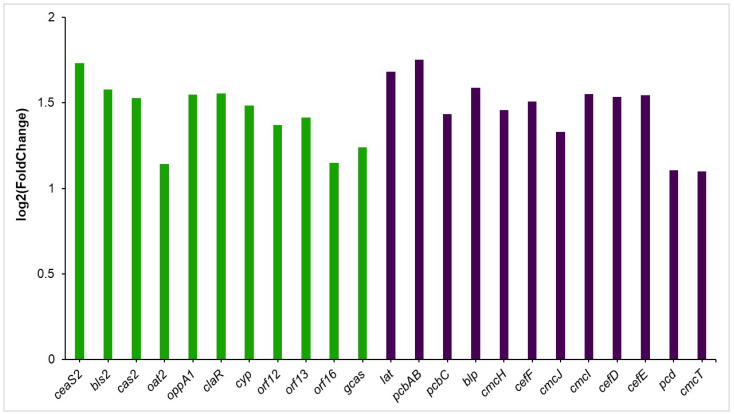
Clavulanic acid and cephamycin C gene cluster expression in *S. clavuligerus* grown in co-culture with *B. velezensis*. Color code: in green, overexpressed CA genes. In purple, cephamycin C overexpressed genes.

Regarding cephamycin C biosynthesis, proteins encoded by up-regulated genes associated with the metabolic pathways of L-2-aminoadipic acid and the amino acids L-cysteine and L-valine were identified; all of them are anabolic precursors of cephamycin C (Figure 6) [24]. Moreover, a significant number of the up-regulated genes are associated with energy production, i.e., ATP synthesis, vitamins such as pyridoxal phosphate, and cofactors. Concerning the down-regulated genes, the expression of genes involved in the synthesis of terpenoid and fatty acids, as well as that of the genes involved in NAD+ synthesis, was decreased in *S. clavuligerus* (Figure 4).

### 3.5. Up-Regulated Genes

#### 3.5.1. Clavulanic Acid and Cephamycin C Clusters

During the co-culture of *S. clavuligerus* with *B. velezensis*, the production of CA increased 2.0-fold, approximately (Figure 1B). This change correlates with the increased transcription rate of most genes within the CA gene cluster (Figure 6). For instance, the *claR* gene, which regulates the late steps of CA biosynthesis, increased its gene transcription rate (1.6-fold). Similarly, genes like *oppA1*, *orf12*, *orf13*, *orf16,* and *gcas*, which are associated with the late steps of the CA metabolic pathway, were significantly over-expressed (Figure 6).

Concerning the genes of the early steps of the CA pathway, it was found that the *ceas2* gene, which encodes the enzyme carboxyethylarginine synthase (it promotes the condensation of L-arginine and glyceraldehyde-3-phosphate (CA precursors) to produce N2-(2-carboxy-ethyl)arginine), had the highest increase in its expression (1.7-fold). Similarly, the gene *bls2*, which encodes the β-lactam synthase, the enzyme that catalyzes the second reaction of the CA metabolic pathway, had a significant improvement in its expression (1.6-fold).

*S. clavuligerus* produces cephamycin C and CA by two different biosynthetic pathways, which are positively regulated by the protein CcaR. To identify the differential metabolic response that could be triggered by *S. clavuligerus* in the presence of *B. velezensis*, the analysis focused on the expression of the cephamycin C gene cluster. It was found that all genes were over-expressed, with the exception of *ccaR* and the *pbp74*, *pbp74,* and *orf10* genes (Figure 6).

To start cephamycin C production, CcaR binds directly to the promoter region of the *lat* gene, activating its transcription [4]. The *lat* gene encodes lysine-amino transferase, the first enzyme of the cephamycin C pathway that forms the α-aminoadipic acid precursor from lysine, in a two-step reaction [24]. In the present study, the *lat* gene expression increased approximately 1.7-fold, and the *pcbAB* gene had an increase in its transcription rate of 1.8-fold. The *pcbAB* gene encodes the δ-(l-α-aminoadipyl)-l-cysteinyl-d-valine synthetase enzyme, one of the first enzymes in the cephamycin C pathway; this enzyme catalyzes the condensation of the three cephamycin C precursor amino acids, α-aminoadipate, cysteine, and valine [5]. Additionally, genes such as *cefF* and *cmcH*, whose primary function is related to reactions of the late steps of cephamycin C, also exhibited a significant increase in their transcription rate (Figure 6).

#### 3.5.2. Differentially Expressed Genes Associated with Stress Response

In this study, the co-culture of *B. velezensis* with *S. clavuligerus* induced the overexpression of genes involved in the neutralization of reactive oxygen species (ROS), e.g., (CRV15_RS08825) and peroxiredoxin (CRV15_RS08820) (2.2- and 1.9-fold, respectively). These two enzymes constitute the alkyl hydroperoxide reductase system reported in other bacteria [25]. Conversely, additional enzymes have been identified as key players in combating oxidative stress, e.g., the heme peroxidase (CRV15_RS24530), whose transcription rate increased by 2.0-fold, and the thioredoxin-dependent thiol peroxidase (CRV15_RS18405), with a 1.1-fold increase in its expression.

Other up-regulated genes associated with different kinds of stress factors were CRV15_RS22875 and CRV15_RS22870. These genes encode an ectoine synthase and an ectoine hydroxylase with a fold change of 1.3 for both cases. Ectoine synthase is the last enzyme in the biosynthetic route for the production of ectoine; ectoine hydroxylase catalyzes the enzymatic conversion of ectoine into 5-hydroxyectoine [26]. In addition, *S. clavuligerus* increased the transcription rate of the genes annotated as CRV15_RS10000, CRV15_RSRS09995, and CRV15_RS12245, which encode the GroES/GroEL chaperonin system; fold change values for these genes were 1.3, 1.6, and 1.5, respectively.

#### 3.5.3. Transcriptional Regulators and Transporters

Many transcriptional regulators were recorded as significant DEGs at 72 h of co-culture (Supplementary Table S2). Remarkably, the gene CRV15_RS11700 was overexpressed (2.0-fold); this gene encodes a response regulator transcription factor, a member of the OmpR family [27], which may have a role in transcriptional regulation of the osmotic stress response [28]. Also, CRV15_RS01435 (which encodes *soxR*) was overexpressed (1.4-fold). *soxR* from *S. clavuligerus* consists of 507 nucleotides and encodes a 189 amino-acid protein which includes a conserved N-terminal HTH DNA-binding site.

Alternatively, the SecD protein encoding *sec*D (CRV15_RS27420) had the highest expression rate (3.7-fold) during co-culture. SecD is part of the sec system, that is, one of the main preprotein secretion routes [29] in Gram-positive bacteria. In this context, sec-dependent secretory proteins are N-terminally tagged with a signal peptide that guides the nascent preprotein to the secretion channel [29]. To evaluate whether among the set of differentially over-expressed genes (DEGs) there were proteins that could be exported by the sec system, the SignalP—6.0 program was used [30]. Table 1 shows five proteins that have a possible sec signal peptide.

#### 3.5.4. Additional Genes Associated with *S. clavuligerus* Metabolism That Were Differentially Expressed During Co-Culture

Two beta-lactamase inhibitor proteins were over-expressed during co-culture: the beta-lactamase inhibitor protein encoded by CRV15_RS04920, and the beta-lactamase inhibitor protein encoded by CRV15_RS06310 (Supplementary Table S2). The latter is part of an operon composed of two other genes, CRV15_RS06305 and CRV15_RS06295, which were over-expressed (1.4 and 1.1-fold, respectively). CRV15_RS06305 encodes the ATP-binding cassette (ABC) domain protein that binds ATP and uses its energy to drive the transport of various molecules across the cell membrane, and CRV15_RS06295, which encodes an ABC transporter permease protein.

Likewise, the biosynthesis of proteinaceous inhibitors that impair subtilisin activity (secreted by *B. velezensis* or as a system required to inhibit its own serine proteases) led to *S. clavuligerus* increasing the transcription rate of CRV15_RS11420 and CRV15_RS31700 by 2.6 and 1.3-fold, respectively. CRV15_RS11420 encodes the subtilase-type protease inhibitor, which inhibited various types of serine proteases, e.g., subtilisin, while CRV15_RS31700 encodes a subtilisin-like protease located in pCLA1.

During co-culture, the expression level of a siderophore protein (CRV15_RS24015) increased 1.6-fold in *S. clavuligerus*, as a response to iron scarcity. Also, several redox-related genes were differentially expressed, e.g., the ferredoxin protein encodes the gene CRV15_RS23895, and increased its expression 1.2-fold. Similarly, two genes expressed from a single operon, CRV15_RS07575 (for another ferredoxin gene) and CRV15_RS07570 (which codes for a cytochrome P450) were 1.1 to 1.5-fold up-regulated.

Concerning the process of oxidative phosphorylation [31], several genes were up-regulated, including genes belonging to the succinate dehydrogenase protein complex of the electron transport chain e.g., CRV15_RS09620 (sdhA, succinate dehydrogenase flavoprotein subunit), CRV15_09625 (succinate dehydrogenase iron-sulfur subunit), CRV15_09610 (sdhC, succinate dehydrogenase) and CRV15_09615 (succinate dehydrogenase hydrophobic membrane anchor subunit); the corresponding fold change is presented in Supplementary Table S1. The electron transport chain is made up of several enzymatic complexes; complex I belongs to the enzyme NADH-ubiquinone oxidoreductase; complex II belongs to the aforementioned succinate dehydrogenase, the cytochrome complex, and the ATPase protein complex [31]. For complex I, genes that encode the NADH-quinone oxidoreductase subunit NuoH (CRV15_RS10640) and the NADH-quinone oxidoreductase subunit G (CRV15_RS10645) were significantly up-regulated (Supplementary Material S1). Likewise, for the complex of cytochrome, the cytochrome c oxidase encoded by CRV15_RS21480 was over-expressed with a 1.4-fold change.

### 3.6. Down-Regulated Genes

During co-culture, all genes belonging to the pyrroloquinoline quinone (PQQ) gene cluster were down-regulated (Supplementary Table S3), as well as CRV15_RS15665; this gene encodes a PQQ-dependent sugar dehydrogenase whose expression decreased 2.0-fold. PQQ serves as a redox cofactor for various bacterial dehydrogenases but is also involved in other biological reactions, such as in the production of polyketide biosynthetic products in various *Streptomyces* [32].

In the context of secondary metabolites, it was observed that the genes annotated as CRV15_RS37320 and CRV15_RS02380 are associated with the sesquiterpenoid and triterpenoid biosynthesis [33], respectively. CRV15_RS37320 encodes a terpene cyclase that is part of a cluster formed by five genes: CRV15_RS00630, CRV15_RS00620, CRV15_RS00615, and CRV15_RS00610. CRV15_RS00620 encodes a cytochrome P450 whose expression was also down-regulated during co-culture. In the case of CRV15_RS02380, it encodes a squalene-hopene cyclase, an important enzyme involved in the biosynthesis of triterpenoids. In addition to terpenoid biosynthesis, holomycin production could also have been affected since the CRV15_RS02205 gene, which encodes the holomycin non-ribosomal peptide synthetase, significantly decreased its expression (1.6-fold).

Meanwhile, the gene CRV15_RS32945 was down-regulated (1.2-fold) during co-culture. It is located in pCLA1 and encodes a type I polyketide synthase. In *S. clavuligerus,* two gene clusters are presumably involved in the biosynthesis of lantibiotics [34]. Lanthipeptides are ribosomally synthesized and post-translationally modified peptide (RiPPs) natural products characterized by the presence of lanthionine and methyllanthionine [35]. During the co-culture of *S. clavuligerus* with *B. velezensis*, the genes CRV15_RS06630 and CRV15_RS06635 decreased their expression (Supplementary Table S3). These two genes encode a lanthionine synthetase and lantibiotic dehydratase, respectively; both presumably are involved in the biosynthesis of lanthionine, an amino acid precursor of lantibiotics [36]. Furthermore, all genes in the cluster showed low expression levels (Supplementary Table S3). The antiSMASH analysis of this cluster (antiSMASH v.7.1.0) predicted that this genome region potentially encodes a lanthipeptide-Class-I. The genes CRV15_RS31175 and CRV15_RS36145, located in the *S. clavuligerus* chromosome pCLA1, are associated with the biosynthesis of a lanthipeptide class IV, similar to the compound venezuelin from *Streptomyces venezuelae* ATCC 10712.

## 4. Discussion

### 4.1. Co-Cultured-Mediated Alterations in Specialized Metabolite Synthesis in S. clavuligerus

The co-culture of *S. clavuligerus* with *B. velezensis* triggered important transcriptional modifications, particularly in the production of secondary metabolites. A significant finding was the up-regulation of genes related to the biosynthesis of CA and cephamycin C, while the expression of genes involved in the production of other specialized metabolites, such as polyketides, terpenoids, and lantibiotics, was down-regulated. The over-expression of many genes of the cephamycin C gene cluster, although not directly quantified at the metabolite level, suggests that co-cultivation may enhance its biosynthesis. Overexpression of beta-lactamase inhibitor proteins, exported via the SecD pathway, further supports a defensive strategy by *S. clavuligerus* to counteract potential threats posed by *B. velezensis*, particularly against beta-lactamase activity. Furthermore, the observed low expression of polyketide, terpene, and lantibiotic biosynthetic pathways might not only reflect the reduction of PQQ-mediated redox activity, as discussed previously, but could also be a consequence of precursor competition under nutrient-limited conditions. In such contexts, *S. clavuligerus* may prioritize the biosynthesis of β-lactam antibiotics (CA and cephamycin C) as key survival metabolites. Thus, the transcriptional response of *S. clavuligerus* appears to reflect an adaptive strategy to optimize its metabolic output under co-culture conditions, enhancing the expression of specific antibiotic biosynthetic pathways (cephamycin C and CA) while down-regulating the expression of other secondary metabolite pathways.

Although the main focus of this study was the transcriptional response of *S. clavuligerus*, an exploratory RNA-Seq analysis of *B. velezensis* was also performed. Despite the absence of biological replicates, this analysis revealed down-regulation of genes related to motility (*flhF*, *fliT*, and *flgC*), iron acquisition (*fbpA*), and beta-lactamase production (RBAM_RS07280) (Supplementary Table S1). These results suggest that *B. velezensis* may experience stress and loss of adaptive defenses under co-culture conditions, potentially driven by nutrient competition, oxidative stress, and exposure to bioactive metabolites produced by *S. clavuligerus*. These observations are consistent with the hypothesis that interspecies interactions dynamically remodel bacterial physiology.

### 4.2. Triggering of Clavulanic Acid Biosynthesis: Role of Oxidative Stress and Peptide Signaling

Our results suggest that the cultivation of *S. clavuligerus* with *B. velezensis* induces the production of endogenous H_2_O_2_ in *S. clavuligerus*, leading to the activation of various defense-related mechanisms. During co-culture, *S. clavuligerus* exhibited increased expression of genes associated with the respiratory chain, which could suggest an attempt to reinforce energy metabolism. However, transcriptomic data also revealed modulation of iron homeostasis genes, including the up-regulation of siderophore-interacting proteins (CRV15_RS07535) (to up-take iron) and repression of Fe-S cluster-related genes (CRV15_RS35455) (to save iron). These findings point toward an iron-limited environment, likely due to competition for iron between the two bacteria in co-culture, which could compromise the functionality of iron-dependent respiratory enzymes. Under such iron stress, even if respiratory chain components are transcriptionally up-regulated, impaired electron flow may occur, resulting in enhanced electron leakage and ROS accumulation [37]. Thus, the observed transcriptional response likely reflects an adaptive strategy to manage oxidative and energy stress rather than a direct increase in ATP production.

Interestingly, the enhanced expression of redox-sensitive regulators such as SoxR, known to respond to superoxide accumulation and to redox-cycling compounds like phenazines, is consistent with the occurrence of oxidative stress. Indeed, the *PhzF* gene (CRV15_RS25190), which encodes a key enzyme in phenazine biosynthesis, and an isochorismatase family cysteine hydrolase gene (CRV15_RS27235) were up-regulated 1.3 and 1.2-fold, respectively, in *S. clavuligerus*. Phenazines are well-known to promote ROS generation and trigger oxidative stress defenses [38,39].

Additionally, *S. clavuligerus* showed increased expression of genes involved in the biosynthesis of ectoine and 5-hydroxyectoine (CRV15_RS22875 and CRV15_RS22870, respectively). Ectoine is a well-recognized osmoprotectant that also stabilizes macromolecules under oxidative stress [40,41]. Its accumulation likely helps *S. clavuligerus* to limit the damage caused by elevated ROS during co-culture.

Together, the activation of the SoxR regulon, the up-regulation of phenazine-related genes, and the production of protective osmolytes such as ectoine, provide evidence that significant oxidative stress occurs in *S. clavuligerus* during co-culture with *B. velezensis*. This stress response likely plays a central role in triggering the biosynthesis of secondary metabolites, including CA and cephamycin C, as part of a survival strategy under competitive conditions.

**Figure 7:**
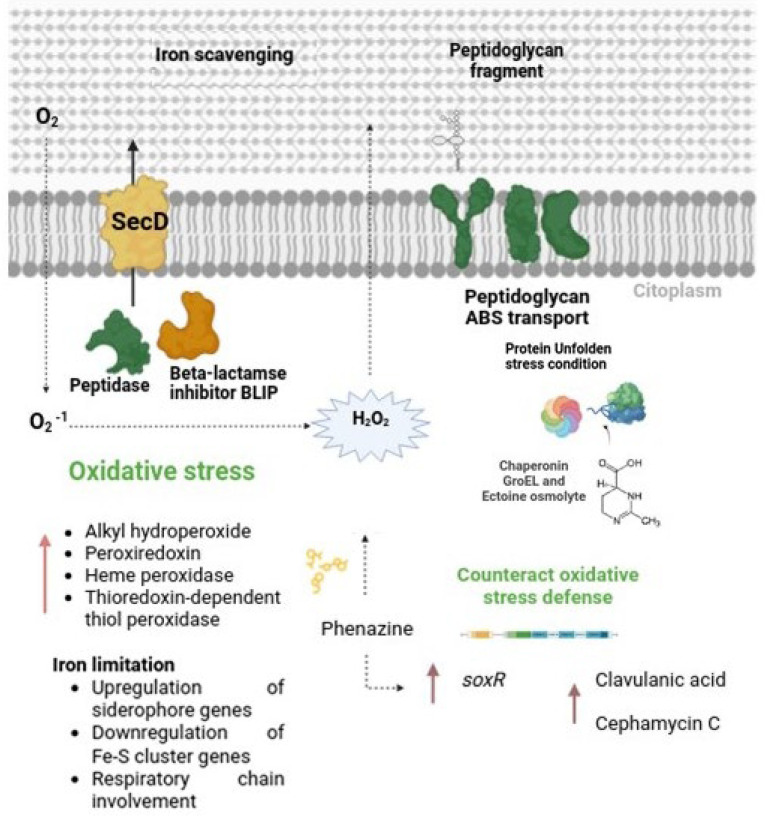
Summary of the most significant effect of co-cultivation on *S. clavuligerus* metabolism. During co-culture, *B. velezensis* induces the accumulation of H_2_O_2_ in *S. clavuligerus*, as well as the possible synthesis of a phenazine-like molecule. The oxidative stress, triggered within *S. clavuligerus,* leads to an increased expression of enzymes responsible for mitigating this stress. Other mechanisms represented, such as peptide signaling and cell-wall sensing, are hypothetical and based on transcriptomic trends. Experimental validation is needed to confirm these interactions. The pink lines correspond to the enzymes, genes, or secondary metabolites positively affected during co-cultivation. The dotted lines correspond to hypothetical interactions.

Additionally, besides oxidative stress, small extracellular peptides may also play a role in the modulation of specialized metabolite production during co-culture. For instance, in *Bacillus* species, peptidoglycan-derived peptides were shown to modulate the expression of a beta-lactamase regulator [42,43]. Therefore, since *S. clavuligerus*, CA and cephamycin C are co-regulated by the activator CcaR, we cannot exclude that peptide fragments originating from impaired cell wall biosynthesis or cell wall degradation may act as signaling molecules and thus also induce the biosynthesis of β-lactam-related compounds. Supporting this idea, we observed up-regulation of a peptidoglycan-binding domain-containing protein (CRV15_RS11635) and associated ABC transporter components (CRV15_RS11630, CRV15_RS11625, and CRV15_RS11640), which may facilitate the internalization of extracellular peptide fragments. Similar mechanisms have been described in *Bacillus* species, where peptidoglycan-derived peptides modulate the expression of beta-lactamase regulator [43]. Although our findings remain speculative, they raise testable hypotheses for future experimental validation. Based on the transcriptomic findings described above, Figure 7 summarizes the potential interactions and regulatory pathways possibly involved in the increased production of CA.

## 5. Conclusions

In this study, we proposed co-cultivation of *S. clavuligerus* with *B. velezensis* as an effective and novel strategy to improve CA production. Furthermore, transcriptomic analysis of the co-culture revealed that *B. velezensis* induces the expression, in *S. clavuligerus,* of antioxidant enzymes that have the ability to reduce oxidative stress generated by a potential H_2_O_2_ accumulation.

Transcriptomic data also revealed modulation of iron homeostasis genes, including the up-regulation of siderophore-interacting proteins (to up take iron) and down-regulation of proteins bearing Fe-S clusters (to save iron). These findings point toward an iron-limited environment due to the co-culture with *B. velezensis* and thus competition for iron between the two bacteria, which could compromise the functionality of iron-dependent respiratory enzymes. Under such iron stress, even if respiratory chain components are transcriptionally up-regulated, impaired electron flow may occur, resulting in enhanced electron leakage and ROS accumulation. Thus, the observed transcriptional response likely reflects an adaptive strategy to manage oxidative and energy stress rather than a direct increase in ATP production. Moreover, it was possible to infer that the expression of several secondary metabolite biosynthetic pathways (terpenes, polyketides, and lantibiotics) were reduced in *S. clavuligerus*, whereas the biosynthesis of cephamycin C and beta-lactamase inhibitor was enhanced in order to counteract *B. velezensis* potential deleterious effects. Notably, co-culture conditions allowed the identification of genes in *S. clavuligerus* whose expression was not previously correlated with CA synthesis, such as those involved in phenazine production.

Finally, it should be noted that elucidating the mechanism by which *B. velezensis* significantly enhances CA production remains a challenge due to the complex interactions of the factors involved.

## Figures and Tables

**Table 1:** Proteins with a sec signal peptide.

Gene Annotation	Gene Product	Signal Peptide (Sec/SPI) Likelihood	Fold-Change
CRV15_RS06310	beta_lactamase_inhibitory_protein_BLIP	0.9089	2.2
CRV15_RS07505	Biotin transporter BioY	0.9804	1.6
CRV15_RS10680	C40 family peptidase	0.8146	2.5
CRV15_RS11710	Hypothetical protein	0.9984	2.6
CRV15_RS29805	Peptidase inhibitor family	0.9943	1.5

## Data Availability

The data presented in this study are available in the Sequence Read Archive (SRA) database in NCBI (Accession No. PRJNA1125460).

## Supplementary Materials

The following supporting information can be downloaded at: https://www.mdpi.com/article/10.3390/metabo15050337/s1, Supplementary Figure S1: KEGG Mapper for some of the most significant up and down regulated genes annotate on KEGG database; Supplementary Material S1: Up-regulated genes; Supplementary Material S2: Down-regulated genes; Supplementary Table S1: Some representative up-regulated genes; Supplementary Table S2: Some representative down-regulated genes. Supplementary Table S3: Some representative down-regulated genes.
