# Supplementary material for: Eliciting Clavulanic Acid Biosynthesis: The Impact of Bacillus velezensis FZB42 on the Metabolism of Streptoyces clavuligerus ATCC 27064
Source: Metabolites. 2025 May 19;15(5):337. doi: 10.3390/metabo15050337 (PMC12113186; doi:10.3390/metabo15050337)
Supplement: Supplementary file 1 [file metabolites-15-00337-s001.zip › Supplementary Figure S1.pdf]

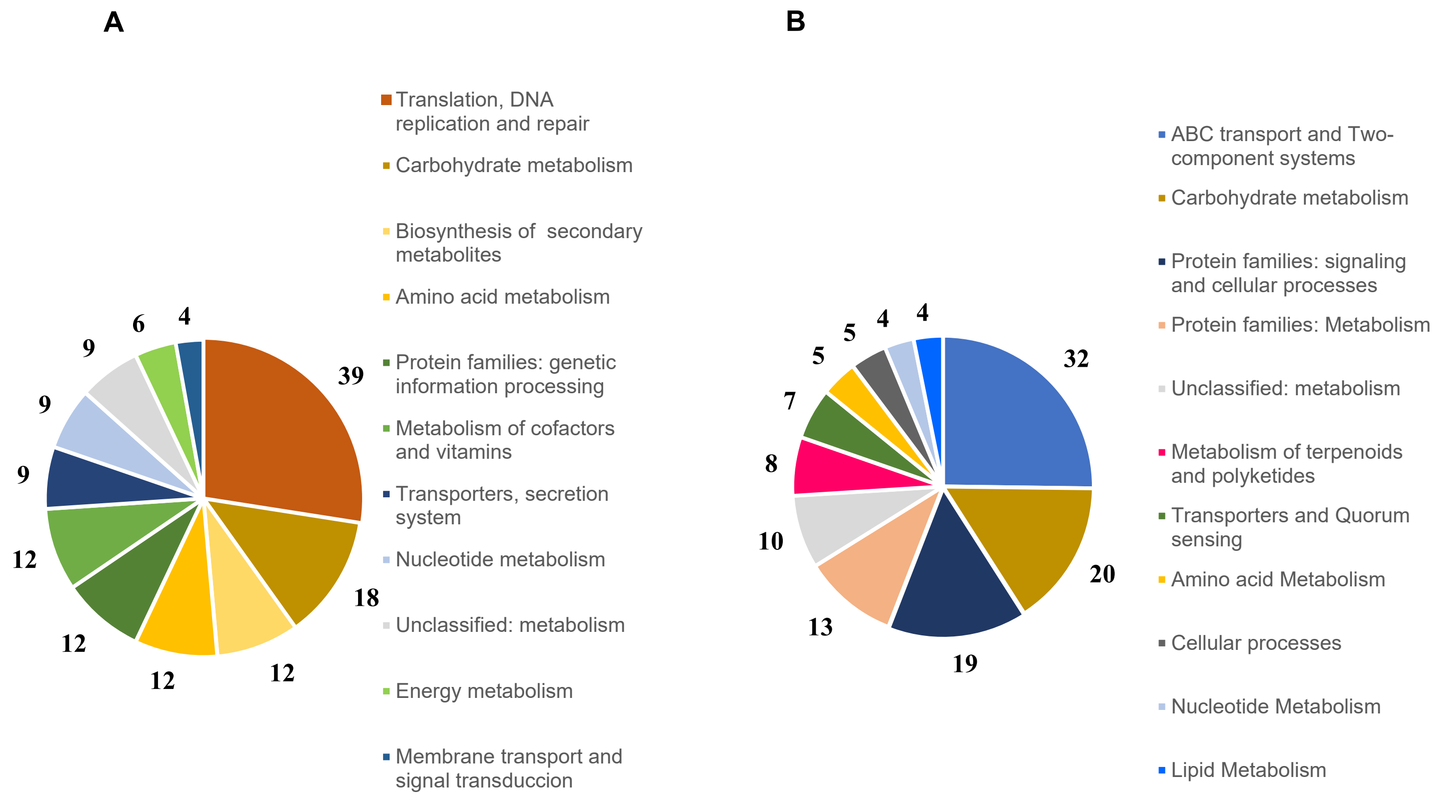

Figure S1. KEGG Mapper for some of the most significant up and down regulated genes annotate on KEGG database. **A)** Up-regulated genes. **B)** Down-regulated genes.
