# Supplementary material for: Eliciting Clavulanic Acid Biosynthesis: The Impact of Bacillus velezensis FZB42 on the Metabolism of Streptoyces clavuligerus ATCC 27064
Source: Metabolites. 2025 May 19;15(5):337. doi: 10.3390/metabo15050337 (PMC12113186; doi:10.3390/metabo15050337)
Supplement: Supplementary file 1 [file metabolites-15-00337-s001.zip › Supplementary Table S1.docx.pdf]

Table S1. Top 20 most affected genes in *Bacillus velezensis* during co-culture with *Streptomyces clavuligerus* at 72 hours. **Data were obtained from a single RNA-Seq experiment without biological replicates and are presented as an exploratory analysis**. Genes are ranked based on log_2 fold change relative to control samples.

Gene	log_2FoldChange	Expression (Treated)	Mean Expression (Control)	Gene product
RBAM_RS19595	-1,04	10	20.5	Hypothetical protein
RBAM_RS10790	-1,04	132	272.0	YjcZ family sporulation protein [Bacillus]
RBAM_RS01245	-1,09	12	25.5	sensor histidine kinase [Bacillus velezensis]
RBAM_RS06650	-1,10	10	21.5	hypothetical protein [Bacillus]
RBAM_RS08190	-1,12	18	39.0	flagellar biosynthesis protein FlhF
RBAM_RS03845	-1,13	22	48.0	aldehyde dehydrogenase family protein
RBAM_RS06655	-1,14	19	42.0	Fur-regulated basic protein FbpA
RBAM_RS16430	-1,17	12	27.0	flagella biosynthesis regulatory protein FliT [Bacillus velezensis]
RBAM_RS04985	-1,20	557	1275.5	YhdX family protein [
RBAM_RS15530	-1,23	10	23.5	copper-sensing transcriptional repressor CsoR [Bacillus]
RBAM_RS17595	-1,24	7	16.5	Hypothetical protein
RBAM_RS06680	-6,15	1	141.5	membrane protein DedA, SNARE-associated domain
RBAM_RS07280	-5,28	21	856.5	Beta-lactamase
RBAM_RS09530	-1,24	15	35.5	N-acetyltransferase [Bacillus]
RBAM_RS17140	-25,31	0	41.5	VWA domain-containing protein [Bacillus]
RBAM_RS01965	-25,06	0	35.0	amino acid ABC transporter substrate-binding protein
RBAM_RS01100	-25,00	0	33.5	2-thiouracil desulfurase family protein, partial [Bacillus velezensis]
RBAM_RS02675	-24,89	0	31.0	Rrf2 family transcriptional regulator [Bacillus]
RBAM_RS08080	-24,74	0	28.0	flagellar basal body rod protein FlgC [
RBAM_RS17280	-24,69	0	27.0	flagellar basal body rod protein FlgC
RBAM_RS16705	-24,58	0	25.0	poly-gamma-glutamate biosynthesis protein PgsC
RBAM_RS15580	-24,42	0	22.5	amino acid ABC transporter ATP-binding protein
RBAM_RS08185	-24,32	0	21.0	flagellar biosynthesis protein FlhA [Bacillus velezensis]
RBAM_RS18110	-24,25	0	20.0	ArsR/SmtB family transcription factor
RBAM_RS17805	-24,14	0	18.5	hypothetical protein
RBAM_RS00930	-24,14	0	18.5	hypothetical protein
RBAM_RS15600	-23,74	0	14.0	amino acid ABC transporter substrate-binding protein
RBAM_RS19590	-23,58	0	12.5	hypothetical protein
RBAM_RS06155	-23,52	0	12.0	hypothetical protein
RBAM_RS07730	-23,52	0	12.0	aspartate carbamoyltransferase catalytic subunit
RBAM_RS01445	-23,52	0	12.0	nitroreductase family protein
RBAM_RS08775	-23,46	0	11.5	hypotetical protein
