# Supplementary material for: Eliciting Clavulanic Acid Biosynthesis: The Impact of Bacillus velezensis FZB42 on the Metabolism of Streptoyces clavuligerus ATCC 27064
Source: Metabolites. 2025 May 19;15(5):337. doi: 10.3390/metabo15050337 (PMC12113186; doi:10.3390/metabo15050337)
Supplement: Supplementary file 1 [file metabolites-15-00337-s001.zip › Supplementary Table S2.pdf]

A selection of representative DEGs

Table S2. Some representative up-regulated genes

Genes	Protein name	log_2FoldChange	*padj*	Reaction or metabolic process
**Energy metabolism**				
CRV15_RS09620	Succinate dehydrogenase flavoprotein subunit, sdhA	1.3	$1.2x10^{-2}$	Citrate cycle (TCA cycle)
CRV15_RS09625	Succinate dehydrogenase iron-sulfur subunit	1.3	$1.3x10^{-2}$	Citrate cycle (TCA cycle)
CRV15_RS09610	Succinate dehydrogenase, sdhC	1.1	$2.8x10^{-2}$	Citrate cycle (TCA cycle)
CRV15_RS09615	Succinate dehydrogenase hydrophobic membrane anchor subunit	1.4	$4.31x10^{-3}$	Citrate cycle (TCA cycle)
CRV15_RS07845	Multifunctional oxoglutarate decarboxylase	1.5	$6.8x10^{-6}$	Citrate cycle (TCA cycle)
CRV15_RS09815	ADP-forming succinate--CoA ligase subunit beta, sucC	1.6	$4.4x10^{-4}$	Citrate cycle (TCA cycle)
CRV15_RS22155	Glutamate synthase large subunit, gltB	1.8	$3.2x10^{-8}$	Nitrogen metabolism
CRV15_RS22160	Glutamate synthase subunit beta	1.9	$1.3x10^{-8}$	Nitrogen metabolism
**Amino acid metabolism**				
CRV15_RS24215	N-acetyl-gamma-glutamyl-phosphate reductase, argC	1.6	$6.1x10^{-5}$	Arginine biosynthesis

CRV15_RS24225	Acetylglutamate kinase, argB	1.3	$6x10^{-4}$	Arginine biosynthesis
CRV15_RS24245	Argininosuccinate lyase, argH	1.3	$7.8x10^{-5}$	Arginine biosynthesis
CRV15_RS07550	Bifunctional glutamate N-acetyltransferase/amino-acid acetyltransferase, argJ	1.1	$2.6x10^{-3}$	Arginine biosynthesis
**Transcriptional regulators**				
CRV15_RS33445	LuxR C-terminal-related transcriptional regulator	2.2	$4.0x10^{-7}$	Not specified
CRV15_RS11700	DNA-binding response regulator, OmpR family	2.0	$4.2x10^{-8}$	Not specified
CRV15_RS02575	WhiB family transcriptional regulator	1.9	$8.0x10^{-8}$	Not specified
CRV15_RS10955	LuxR C-terminal-related transcriptional regulator	1.6	$1.4x10^{-5}$	Not specified
CRV15_RS07560	LysR family transcriptional regulator	1.6	$3.1x10^{-7}$	Not specified
CRV15_RS05115	nrdR transcriptional regulator NrdR	1.3	$1.4x10^{-3}$	Not specified
CRV15_RS14055	PadR family transcriptional regulator	1.3	$2.x10^{-4}$	Not specified
CRV15_RS14470	TetR/AcrR family transcriptional regulator	1.1	$2.8x10^{-3}$	Not specified
CRV15_RS14460	Response regulator transcription factor	1.1	$1.1x10^{-2}$	Not specified
CRV15_RS11150	GntR family transcriptional regulator	1.0	$3.5x10^{-5}$	Not specified
**Miscellaneous genes**				
CRV15_RS27420	Protein translocase subunit, SecD	3.7	$1.2x10^{-11}$	Bacterial secretion system
CRV15_RS02865	Potassium-transporting ATPase subunit KdpA	1.7	$6.7x10^{-6}$	Two-component system

CRV15_RS02855	Potassium-transporting ATPase subunit C	1.9	$9.3x10^{-7}$	Two-component system
CRV15_RS02860	Potassium-transporting ATPase subunit KdpB	1.5	$2.9x10^{-5}$	Two-component system
CRV15_RS04920	Beta-Lactamase Inhibitor *Proteins (BLIP)*	2.5	$8.2x10^{-13}$	Antimicrobial resistance.
CRV15_RS06310	Beta-lactamase inhibitory protein (BLIP)	2.2	$2.9x\text{-}10^{-3}$	Antimicrobial resistance
CRV15_RS08825	Alkyl hydroperoxide reductase	2.2	$8.8x10^{-4}$	Detoxification of reactive oxygen species
CRV15_RS18405	Thioredoxin-dependent thiol peroxidase	1.1	$1.6x10^{-2}$	Detoxification of reactive oxygen species
CRV15_RS24530	Heme peroxidase family protein	2.0	$6.5x10^{-5}$	Detoxification of reactive oxygen species
CRV15_RS08820	Peroxiredoxin	1.9	$5.5x10^{-5}$	Detoxification of reactive oxygen species
CRV15_RS23405	Transglycosylase family protein	3.6	$8.5x10^{-3}$	Bacterial cell wall remodeling
CRV15_RS	PhzF phanazine biosynthesis protein	1.2	$7x10^{-3}$	Secondary metabolism

Table S2. Some representative down-regulated genes

Miscellaneous genes				
**Genes**	**Protein name**	**log_2FoldChange**	***padj***	**Reaction or metabolic process**
CRV15_RS00255	Terpene synthase family protein	-1.7	$5.5x10^{-3}$	Terpenoid backbone biosynthesis
CRV15_RS37550	Terpene synthase family protein	-2.8	$1.4x10^{-7}$	Terpenoid backbone biosynthesis
CRV15_RS02380	shc squalene-hopene cyclase	-1.1	$7.4x10^{-3}$	Sesquiterpenoid and triterpenoid biosynthesis
CRV15_RS34070	Enediyne biosynthesis protein E4	-1.8	$6.1x10^{-5}$	Biosynthesis of enediyne antibiotics
CRV15_RS34075	Enediyne biosynthesis protein UnbU	-1.3	$2.4x10^{-2}$	Biosynthesis of enediyne antibiotics

CRV15_RS06625	Thiopeptide-type bacteriocin protein	-1.2	$8.9x10^{-4}$	Peptide antibiotics
CRV15_RS06630	Lanthionine synthetase C family protein	-1.5	$6.3\ x10^{-5}$	Lanthionine biosynthesis
CRV15_RS06635	Lantibiotic dehydratase	-1.3	$8.0x10^{-5}$	Lantibiotic biosynthesis
CRV15_RS06640	FxLD family lanthipeptide	-1.6	$1.7x10^{-6}$	Lanthipeptide biosynthesis
CRV15_RS06645	FxlM methyltransferase	-1.2	$3.2x10^{-3}$	Lanthipeptide biosynthesis
CRV15_RS06650	ATP-binding protein	-1.4	$3.9x10^{-4}$	
CRV15_RS31175	lanL class IV lanthionine synthetase LanL	-2.2	$1.3x10^{-12}$	Lanthionine biosynthesis
CRV15_RS36145	VenA family class IV lanthipeptide	-2.2	$4.1x10^{-7}$	Lanthipeptide biosynthesis
CRV15_RS03040	PQQ-dependent sugar dehydrogenase	-3.0	$3.0x10^{-19}$	Pyrroloquinoline biosynthesis
CRV15_RS03070	Pyrroloquinoline-quinone synthase PqqC	-2.1	$1.7x10^{-08}$	Pyrroloquinoline biosynthesis
CRV15_RS03060	pyrroloquinoline quinone biosynthesis protein PqqE	-2.1	$1.5x10^{-8}$	Pyrroloquinoline biosynthesis
CRV15_RS03080	pyrroloquinoline quinone precursor peptide PqqA	-1.9	3.6x10-7	Pyrroloquinoline biosynthesis
CRV15_RS37550	Terpene synthase family protein	-2.8	$1.4\ x10^{-7}$	Terpene biosynthesis
CRV15_RS24045	Gamma-glutamyl-gamma-aminobutyrate hydrolase	-1.7	$1.4x10^{-7}$	Amine and polyamine degradation
**Transcriptional regulators**				
CRV15_RS18950	MerR	-1.8	$1.18219\ x10^{-7}$	Not specified
CRV15_RS19040	MarR winged hélix-turn-helix	-1.4	$1.23x10^{-2}$	Not specified
CRV15_RS08145	Fur	-1.2	$5.6x10^{-4}$	Not specified
CRV15_RS21130	TetR	-1.2	$1.9x10^{-4}$	Not specified
CRV15_RS04445	PaaX	-1.0	$1.905x10^{-4}$	Not specified
CRV15_RS03000	ROK	-1.0	$8.0x10^{-2}$	Not specified
CRV15_RS20765	DeoR/GlpR	-1.0	$6.2x10^{-3}$	Not specified
