# Supplementary material for: Eliciting Clavulanic Acid Biosynthesis: The Impact of Bacillus velezensis FZB42 on the Metabolism of Streptoyces clavuligerus ATCC 27064
Source: Metabolites. 2025 May 19;15(5):337. doi: 10.3390/metabo15050337 (PMC12113186; doi:10.3390/metabo15050337)
Supplement: Supplementary file 1 [file metabolites-15-00337-s001.zip › Table S3.pdf]

A selection of representative DEGs

Table S3. Some representative down-regulated genes

**Miscellaneous genes**				
**Genes**	**Protein name**	**log_2FoldChange**	***padj***	**Reaction or metabolic process**
CRV15_RS00255	Terpene synthase family protein	-1.7	$5.5x10^{-3}$	Terpenoid backbone biosynthesis
CRV15_RS37550	Terpene synthase family protein	-2.8	$1.4x10^{-7}$	Terpenoid backbone biosynthesis
CRV15_RS02380	shc squalene-hopene cyclase	-1.1	$7.4x10^{-3}$	Sesquiterpenoid and triterpenoid biosynthesis
CRV15_RS34070	Enediyne biosynthesis protein E4	-1.8	$6.1x10^{-5}$	Biosynthesis of enediyne antibiotics
CRV15_RS34075	Enediyne biosynthesis protein UnbU	-1.3	$2.4x10^{-2}$	Biosynthesis of enediyne antibiotics
CRV15_RS06625	Thiopeptide-type bacteriocin protein	-1.2	$8.9x10^{-4}$	Peptide antibiotics
CRV15_RS06630	Lanthionine synthetase C family protein	-1.5	$6.3 x10^{-5}$	Lanthionine biosynthesis
CRV15_RS06635	Lantibiotic dehydratase	-1.3	$8.0x10^{-5}$	Lantibiotic biosynthesis
CRV15_RS06640	FxLD family lanthipeptide	-1.6	$1.7x10^{-6}$	Lanthipeptide biosynthesis
CRV15_RS06645	FxlM methyltransferase	-1.2	$3.2x10^{-3}$	Lanthipeptide biosynthesis
CRV15_RS06650	ATP-binding protein	-1.4	$3.9x10^{-4}$	
CRV15_RS31175	lanL class IV lanthionine synthetase LanL	-2.2	$1.3x10^{-12}$	Lanthionine biosynthesis
CRV15_RS36145	VenA family class IV lanthipeptide	-2.2	$4.1x10^{-7}$	Lanthipeptide biosynthesis
CRV15_RS03040	PQQ-dependent sugar dehydrogenase	-3.0	$3.0x10^{-19}$	Pyrroloquinoline biosynthesis

CRV15_RS03070	Pyrroloquinoline-quinone synthase PqqC	-2.1	$1.7x10^{-08}$	Pyrroloquinoline biosynthesis
CRV15_RS03060	pyrroloquinoline quinone biosynthesis protein PqqE	-2.1	$1.5x10^{-8}$	Pyrroloquinoline biosynthesis
CRV15_RS03080	pyrroloquinoline quinone precursor peptide PqqA	-1.9	3.6x10-7	Pyrroloquinoline biosynthesis
CRV15_RS37550	Terpene synthase family protein	-2.8	$1.4\ x10^{-7}$	Terpene biosynthesis
CRV15_RS24045	Gamma-glutamyl-gamma-aminobutyrate hydrolase	-1.7	$1.4x10^{-7}$	Amine and polyamine degradation
**Transcriptional regulators**				
CRV15_RS18950	MerR	-1.8	$1.18219\ x10^{-7}$	Not specified
CRV15_RS19040	MarR winged hélix-turn-helix	-1.4	$1.23x10^{-2}$	Not specified
CRV15_RS08145	Fur	-1.2	$5.6x10^{-4}$	Not specified
CRV15_RS21130	TetR	-1.2	$1.9x10^{-4}$	Not specified
CRV15_RS04445	PaaX	-1.0	$1.905x10^{-4}$	Not specified
CRV15_RS03000	ROK	-1.0	$8.0x10^{-2}$	Not specified
CRV15_RS20765	DeoR/GlpR	-1.0	$6.2x10^{-3}$	Not specified
